# Mitochondrial sirtuins, key regulators of aging

**DOI:** 10.1093/lifemedi/lnaf019

**Published:** 2025-06-09

**Authors:** Zhejun Ji, Guang-Hui Liu, Jing Qu

**Affiliations:** State Key Laboratory of Organ Regeneration and Reconstruction, Institute of Zoology, Chinese Academy of Sciences, Beijing 100101, China; Beijing Institute for Stem Cell and Regenerative Medicine, Beijing 100101, China; State Key Laboratory of Organ Regeneration and Reconstruction, Institute of Zoology, Chinese Academy of Sciences, Beijing 100101, China; Beijing Institute for Stem Cell and Regenerative Medicine, Beijing 100101, China; University of Chinese Academy of Sciences, Beijing 100049, China; Advanced Innovation Center for Human Brain Protection, National Clinical Research Center for Geriatric Disorders, Xuanwu Hospital Capital Medical University, Beijing 100053, China; Aging Biomarker Consortium (ABC), Beijing 100101, China; State Key Laboratory of Organ Regeneration and Reconstruction, Institute of Zoology, Chinese Academy of Sciences, Beijing 100101, China; Beijing Institute for Stem Cell and Regenerative Medicine, Beijing 100101, China; University of Chinese Academy of Sciences, Beijing 100049, China; Advanced Innovation Center for Human Brain Protection, National Clinical Research Center for Geriatric Disorders, Xuanwu Hospital Capital Medical University, Beijing 100053, China; Beijing Institute of Heart Lung and Blood Vessel Diseases, Beijing Anzhen Hospital, Capital Medical University, Beijing 100029, China

**Keywords:** mitochondrial sirtuins, mitochondrial dysfunction, aging

## Abstract

Mitochondrial dysfunction is a hallmark of aging, characterized by a decline in mitochondrial biogenesis and quality control, compromised membrane integrity, elevated ROS production, damaged mitochondrial DNA (mtDNA), impaired mitochondrial–nuclear crosstalk, and deregulated metabolic balance. Among the key longevity regulators, sirtuin family members SIRT3, SIRT4, and SIRT5 are predominantly localized to mitochondria and play crucial roles in maintaining mitochondrial function and homeostasis. This review explores how mitochondrial sirtuins mitigate aging-related mitochondrial dysfunctions and their broader implications in aging-related diseases. By elucidating the intricate interplay between mitochondrial dysfunction and mitochondrial sirtuins, we aim to provide insights into therapeutic strategies for promoting healthy aging and combating age-related pathologies.

## Introduction

Aging is an intricate, multi-dimensional biological process marked by the progressive deterioration of bodily functions and heightened vulnerability to disease and mortality [1, 2]. It affects all living organisms and is driven by a combination of genetic, environmental, and lifestyle factors [3, 4]. The manifestations of aging include the accumulation of cellular and molecular damage, loss of tissue homeostasis, and impairment of regenerative capacity, which together contribute to the increased risk of developing age-related diseases, such as cardiovascular diseases, neurodegenerative disorders, cancer, and metabolic syndromes [5, 6]. Understanding the mechanisms underlying aging is essential for identifying potential interventions that could promote healthy aging and longevity. The “biomarkers of aging” framework categorizes the key features of the aging process, providing a comprehensive understanding of the biological processes that drive aging. These biomarkers include mitochondrial dysfunction, metabolic changes, chronic inflammation, genomic instability, epigenetic alteration, cellular senescence, telomere attrition, proteostatic stress, and others [5, 7–9].

Mitochondria, as cellular powerhouses and biosynthesis centers, is intrinsically linked to metabolic regulation and protein homeostasis, thus playing a pivotal role in the aging process [10]. The dysfunction of mitochondria, a hallmark of aging, is characterized by dysregulated ATP production, increased ROS generation, impaired mitochondrial biogenesis, and other related phenomena [11]. Mitochondrial dysfunction often leads to metabolic changes and increased production of ROS. Elevated ROS, in turn, promotes genomic instability by damaging DNA and induces epigenetic changes that alter gene expression patterns [12]. Similarly, ROS and metabolic disturbances can contribute to proteostatic stress by impairing protein folding and degradation processes. Furthermore, telomere attrition and genomic instability drive cells toward a senescent state, exacerbating chronic inflammation and further aggravation of cellular senescence [3, 13]. In summary, mitochondrial dysfunction lies at a core of aging, acting as a central nexus that links metabolic dysregulation, genomic instability, proteostatic stress, and cellular inflammation to the progression of age-related decline.

Sirtuins, colloquially termed “longevity proteins,” are central regulators in the intricate molecular networks of aging. These proteins function as nicotinamide adenine dinucleotide (NAD^+^)-dependent deacetylases or adenosine diphosphate (ADP)-ribosyltransferases, operating within multiple cellular regulatory pathways crucial to the aging process [14]. The mammalian sirtuin family comprises seven members (SIRT1–7), with SIRT3, SIRT4, and SIRT5 specifically localized to the mitochondria. These mitochondrial sirtuins have garnered significant scientific interest due to their potential roles in aging and age-associated disorders, primarily through their involvement in maintaining mitochondrial function and energy metabolism [15]. Through the regulation of mitochondrial metabolism, stress response pathways, and other cellular processes, these proteins contribute to the maintenance of mitochondrial integrity and function, thereby supporting overall cellular homeostasis [15].

The diverse actions of mitochondrial sirtuins contribute to delaying age-related functional decline in various organs and extending lifespan in model organisms, positioning them as central players in the complex biology of aging. Given their critical roles in regulating aging, a systematic review of SIRT3, SIRT4, and SIRT5 functions in aging and age-related diseases is warranted. This review aims to provide a comprehensive overview of the current understanding of mitochondrial sirtuins, focusing on their involvement in various aging processes and their roles in age-related pathologies.

## Regulatory function of mitochondrial sirtuins

**Figure 1.**
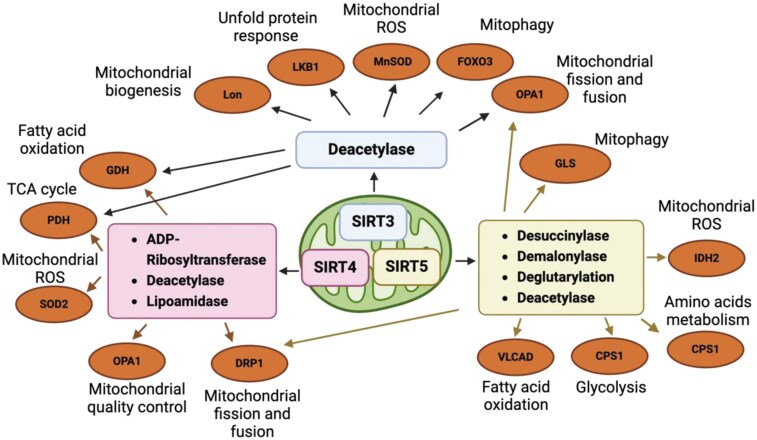
**Mitochondrial sirtuins-associated protein modifications.**Mitochondrial sirtuins, including SIRT3, SIRT4, and SIRT5, exhibit distinct catalytic functions for post-translational modification of proteins that are crucial for maintaining mitochondrial homeostasis. SIRT3 primarily functions as a deacetylase, regulating the activity of key metabolic enzymes involved in TCA cycle, mitochondrial quality control, antioxidant defense, and more. SIRT4 acts as an ADP-ribosyltransferase and deacetylase, modulating mitochondrial ROS, and mitochondrial quality, while also participating in lipid metabolism regulation. SIRT5 possesses demalonylase, desuccinylase, deglutarylase, and deacetylase activities, which influence metabolic pathways such as fatty acid oxidation, amino acid metabolism, and ROS regulation.

Mitochondrial sirtuins exhibit distinct catalytic functions that are crucial for maintaining mitochondrial homeostasis (Fig. 1). Among them, SIRT3 is the most studied mitochondrial sirtuin and plays a critical role in maintaining mitochondrial function. It primarily acts as a deacetylase, removing acetyl groups from various mitochondrial proteins to regulate their activity [16]. SIRT3 is involved in several key mitochondrial events, including promoting mitochondrial biogenesis through the AMP-activated protein kinase (AMPK) and PPAR-Gamma-Coactivator-1α (PGC-1α) pathway, promoting mitochondrial quality control via mitochondrial unfolded protein responses (mtUPR) regulation [16, 17]. In addition, SIRT3 deacetylates key metabolic enzymes in the tricarboxylic acid (TCA) cycle, electron transport chain (ETC), and fatty acid oxidation pathway. These deacetylation events fine-tune metabolic pathways, ensuring optimal energy production and utilization [18]. Furthermore, SIRT3 plays a crucial role in maintaining cellular redox balance. It enhances antioxidant enzymes like superoxide dismutase 2 (SOD2), reducing oxidative stress and protecting cells from mtDNA damage [19]. Beyond its mitochondrial functions, SIRT3 also regulates gene expression and epigenetic modifications outside the mitochondria, influencing cellular responses to stress and metabolic regulation [15].

SIRT4 has a more diverse range of functions compared to SIRT3, although it is less characterized. For example, SIRT4 has been associated with several enzymatic functions, including ADP-ribosylation of glutamate dehydrogenase (GDH) during fatty acid oxidation, lipoamidase activity targeting the pyruvate dehydrogenase (PDH) complex for TCA cycle regulation, and lysine deacetylation involved in regulating leucine metabolism [20]. Additionally, over-expression of SIRT4 was associated with the regulation of mitochondrial dynamics through the inhibition of extracellular signal-regulated kinases (ERK)-mediated phosphorylation of dynamin-related protein 1 (DRP1), a pro-fission factor of mitochondria. By blocking DRP1 phosphorylation, SIRT4 inhibits its activity and subsequently suppresses mitochondrial fission [21]. Moreover, increased endogenous expression of SIRT4 suppresses mitochondrial membrane potential, elevates mitochondrial ROS (mtROS) levels, and induces changes in mitochondrial morphology, leading to mitochondrial aggregation [22]. SIRT4 has also been shown to influence insulin secretion and lipid metabolism, making it relevant to conditions, such as diabetes and obesity [15]. Furthermore, SIRT4 plays a role in the cellular response to stress and nutrient availability, and its dysfunction has been linked to metabolic imbalances and potentially to accelerated aging [15].

SIRT5 is unique among the mitochondrial sirtuins because it exhibits demalonylase, desuccinylase, and deglutarylase, as well as deacetylase activities, which allow it to remove specific acyl modifications from mitochondrial proteins [15]. SIRT5 overexpression leads to an increase in mitochondrial size and a decrease in mitophagy [23]. By contrast, the deletion of *Sirt5* leads to the accumulation of DRP1 in mitochondria and promotes mitochondrial fission in mouse embryonic fibroblasts [24]. Specifically, SIRT5 plays a critical role in regulating various metabolic processes, including glucose metabolism and glycolysis, fatty acid oxidation, amino acid degradation, and the maintenance of ROS homeostasis. Its involvement in these pathways suggests that dysregulation of SIRT5 is associated with to the progression of several diseases, such as metabolic disorders, cardiovascular and neurodegenerative conditions, infectious diseases, and cancer [25].

Although all are located in the mitochondrial matrix, SIRT3, SIRT4, and SIRT5 have distinct functions and display different enzymic modification mechanisms. In some cases, they work synergistically, while under other conditions they act antagonistically [15]. For example, all mitochondrial sirtuins have been reported to inhibit mitochondrial fission via blocking DRP1 activity [21, 24, 26]. By contrast, SIRT3 and SIRT5 were shown to reduce ROS levels, while SIRT4 enhanced it [15]. Moreover, during caloric restriction (CR), SIRT3 and SIRT5 enhance fatty acid oxidation, while SIRT4 counteracts their effects by inhibiting GDH activity through ADP-ribosylation [15]. In summary, SIRT3, SIRT4, and SIRT5 play critical roles in various aspects of mitochondrial function, and their changes with aging are central to the development of mitochondrial dysfunction. In the following sections, we will explore the specific roles of mitochondrial sirtuins in each of the mitochondrial dysfunction during aging, and some aging hallmarks, highlighting their potential impact on the aging process and the development of age-related disease.

## Mitochondrial sirtuins in age-related mitochondrial dysfunction

**Figure 2.**
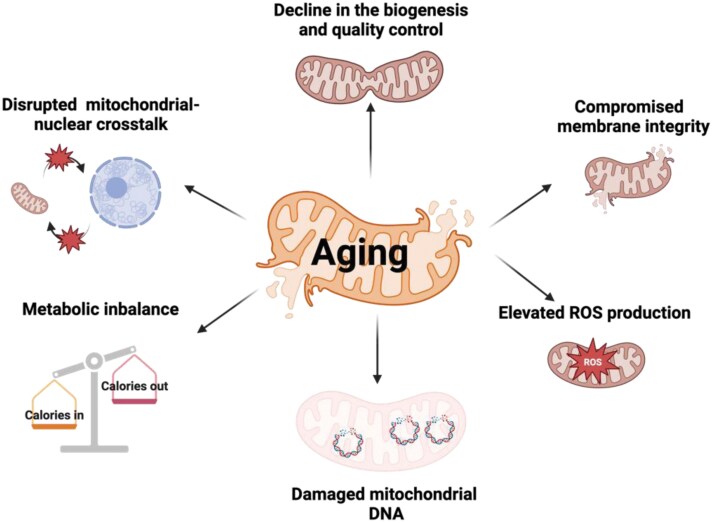
**Mitochondrial dysfunctions during aging.**Age-related mitochondrial dysfunction manifests through multiple alterations: decline in the quality and biogenesis control, compromised membrane integrity, elevated ROS production, damaged mtDNA, disrupted mitochondrial–nuclear crosstalk, and metabolic imbalance. These perturbations drive cellular senescence and age-associated pathologies, underscoring mitochondrial homeostasis as a critical determinant in aging progression.

**Figure 3.**
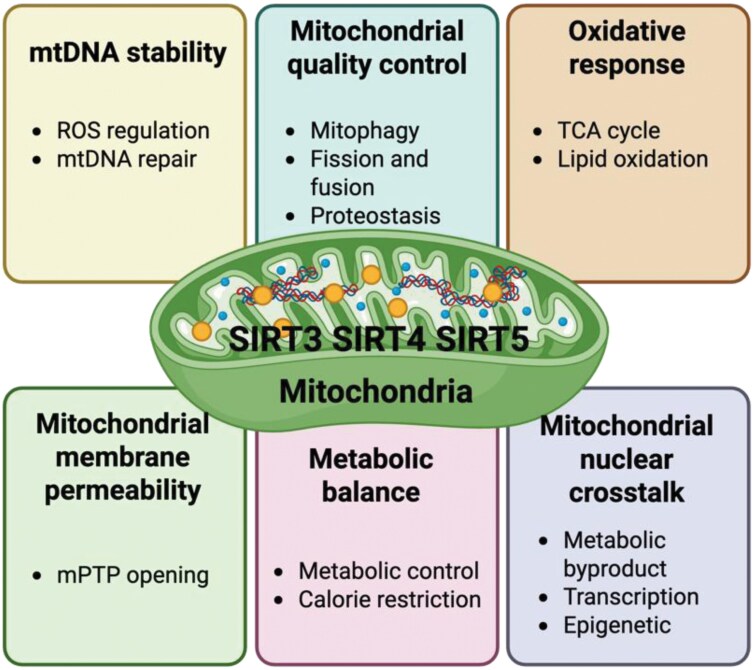
**Mitochondrial sirtuins' roles in mitochondrial functional maintenance.**SIRT3, SIRT4, and SIRT5 play critical roles in mitigating mitochondrial dysfunction during aging. They are involved in maintaining mtDNA stability, regulating mitochondrial quantity and quality, preserving mitochondrial membrane integrity, reducing mtROS levels, facilitating mitochondrial–nuclear communication, and supporting nutrient sensing pathways. These functions collectively highlight their importance in safeguarding mitochondrial health and cellular homeostasis during the aging process.

As discussed above, mitochondrial function emerges as a central regulator in aging. Dysfunctional mitochondria disrupt metabolic pathways, elevate oxidative stress, and propagate a cascade of age-associated damage, affecting nearly every aspect of cellular health. During aging, mitochondria exhibit a range of dysfunctions, including a decline in both their biogenesis ability and quality control, compromised membrane integrity, elevated ROS production, damaged mitochondrial DNA (mtDNA), impaired mitochondrial-nuclear crosstalk, and deregulated metabolic balance (Fig. 2). Of note, SIRT3, SIRT4, and SIRT5 have distinct core functions within mitochondria. With aging, changes in their expression levels or catalytic activities may lead to different types of mitochondrial dysfunction, either accelerating or slowing the aging process. In the following sections, we will summarize each type of aging-related mitochondrial dysfunction and the key roles of mitochondrial sirtuins involved in these processes (Fig. 3).

### Mitochondrial sirtuins in aging-related decline of mitochondrial biogenesis

The changes in mitochondrial numbers with aging remain controversial, with different studies reporting varying trends. In some tissues, such as skeletal muscle, liver, and heart, mitochondrial numbers typically decline with age due to impaired biogenesis, increased oxidative damage, and metabolic imbalance [27–29]. However, studies on rat cardiomyocytes have shown that mitochondrial volume may remain unchanged during aging [30, 31]. Conversely, in certain tissues such as blood cells, mitochondrial quantity may actually increase with aging as a compensatory response. This rise in mitochondrial number is driven by enhanced mitochondrial fission and reduced mitophagy, although these mitochondria may be functionally compromised [32]. Despite these variations, it is widely accepted that mitochondrial biogenesis declines with age.

Mitochondrial biogenesis, the process of generating new mitochondria, is essential for maintaining cellular energy levels and supporting metabolic function, particularly in tissues with high energy demands, such as muscle, heart, and brain [33]. It involves the coordinated expression of nuclear and mitochondrial genes, replication of mtDNA, and the synthesis and import of proteins required for oxidative phosphorylation (OXPHOS). Central to this process is PGC-1α, the master regulator of mitochondrial biogenesis. PGC-1α controls the expression of genes involved in OXPHOS, stimulates mtDNA replication, and enhances mitochondrial function and metabolism [34]. However, during aging, the expression and activity of PGC-1α are significantly reduced, leading to impaired mitochondrial biogenesis [35]. For instance, several studies have shown that lower PGC-1α expression correlates with reduced mtDNA levels and mitochondrial density in skeletal muscles of aged rodents and humans [36]. By contrast, PGC-1α overexpression in muscle has been shown to protect aging mice from age-related muscle wasting and glucose intolerance [36]. The regulation of PGC-1α is mediated by the AMPK pathway and SIRT1, both of which are compromised during aging, further exacerbating the decline in mitochondrial biogenesis [37].

SIRT3 is also a key regulator of mitochondrial biogenesis [38]. It activates AMPK by increasing the AMP/ATP ratio, leading to the phosphorylation of PGC-1α and enhancing SIRT1 activity via elevated NAD^+^ levels. This cascade promotes mitochondrial biogenesis and has been shown to slow the progression of neurodegenerative diseases such as Alzheimer’s disease (AD) [38, 39]. However, both the protein levels and enzymatic activity of SIRT3 decline with age in mammals [40]. This age-related decline in SIRT3 disrupts its diverse functions, contributing to impaired mitochondrial biogenesis and quality control. Specifically, SIRT3-mediated deacetylation of PGC-1α facilitates its interaction with nuclear respiratory factors 1 and 2 (NRF1/2), which in turn activate mitochondrial transcription factor A (TFAM). This process promotes the synthesis and import of nuclear-encoded ETC complex subunits, such as succinate dehydrogenase (SDH) and cytochrome c oxidase. Additionally, SIRT3 regulates Lon proteases (Lon), which selectively degrade TFAM, thereby influencing mitochondrial biogenesis [41]. However, in several age-related disease models, including pre-diabetes, cardiac hypertrophy, and atherosclerosis, the SIRT3/PGC-1α axis is disrupted, leading to diminished respiratory capacity and increased oxidative stress [16].

In contrast to SIRT3, the roles and mechanisms of SIRT4 and SIRT5 in mitochondrial biogenesis are less well understood. Emerging evidence suggests that SIRT4 may modulate the AMPK/PGC-1α pathway by regulating the AMP/ATP ratio, thereby influencing mitochondrial biogenesis [42]. As for SIRT5, studies have shown that its overexpression during bovine preadipocyte differentiation upregulates the mRNA levels of key mitochondrial biogenesis-related genes, including carnitine palmitoyltransferase 1A (CPT1A), NRF1, PGC-1α, and TFAM [43]. Collectively, these findings underscore that the age-related decline in mitochondrial biogenesis regulators compromises both mitochondrial quantity and function. While SIRT3 is well-established as a central player in mitochondrial biogenesis, SIRT4 and SIRT5 may also contribute to this process, albeit through less clearly defined mechanisms.

### Mitochondrial sirtuins in aging-related impairment of mitochondrial quality control

As organisms age, not only is the biogenesis of mitochondria affected, but also more notably, mitochondrial quality declines significantly, leading to the accumulation of dysfunctional mitochondria and contributing to cellular deterioration and the development of age-related diseases [11]. The quality of mitochondria is strictly regulated by a series of quality control mechanisms, including the mtUPR, mitophagy, and regulation of mitochondrial dynamics (fission and fusion) [44].

#### Mitochondrial sirtuins in mtUPR

The mtUPR is a stress-response mechanism that activates the transcription of genes encoding mitochondrial chaperones and proteases, such as heat shock protein 60 (Hsp60) and heat shock protein 10 (Hsp10). It is a vital process that ensures the proper folding, assembly, and degradation of mitochondrial proteins that help maintain protein stability and functionality within the mitochondria [45]. During aging, in response to mtDNA mutation accumulation, ROS interference, and impaired mitochondrial–nuclear communication, the mtUPR is activated to restore the proper function of mitochondria [46]. Many studies have shown that activating the mtUPR can promote lifespan extension in *C. elegans*, *D. melanogaster*, and mice, indicating a conserved function in sustaining cellular homeostasis over the long term [47, 48].

SIRT3 modulates mtUPR by ensuring the proper function of molecular chaperones and proteases involved in the refolding or degradation of misfolded proteins. Specifically, activation of AMPK, potentially through SIRT3-mediated deacetylation of liver kinase B1 (LKB1), has been shown to increase the expression of mtUPR-related genes, such as lon peptidase 1 (LONP1) and HSP60 [49, 50]. LONP1, a serine protease, selectively targets misfolded, incomplete, or oxidized proteins in the mitochondrial matrix for degradation [50]. HSP60 is a chaperone protein that facilitates the folding of polypeptides into functional proteins and complexes and also prevents protein aggregation under stress conditions [50]. Additionally, SIRT3 deacetylates several critical mitochondrial proteins, including manganese superoxide dismutase (MnSOD), HSP10, and LONP1, which are involved in protecting mitochondria from oxidative stress and ensuring proper protein folding through the mtUPR [51–53].

By contrast, SIRT4 has not been directly implicated in regulating mtUPR. However, it may influence mitochondrial ROS levels and mtDNA stability, indirectly affecting the mtUPR. Meanwhile, SIRT5’s deacylation activity is crucial for maintaining the proper function of enzymes involved in the TCA cycle and oxidative phosphorylation, ensuring efficient mitochondrial energy production and preventing metabolic imbalances that could lead to dysfunction. When SIRT5 is silenced, succinylation of glutaminase (GLSN)—a key enzyme that converts glutamine into glutamate for ammonia production in mitochondria—increases. This process also elevates the expression of autophagy and mitophagy markers, including LC3 paralogs, BNIP3, and the PTEN-induced putative kinase 1 (PINK1)–Parkin pathway, in human breast cancer cell lines and mouse myoblasts [23].

#### Mitochondrial sirtuins in mitophagy

When misfolded proteins accumulate beyond the capacity of the mtUPR, mitophagy appears to alleviate mitochondrial dysfunction. Mitophagy is a critical process for selectively degrading damaged mitochondria, thereby preventing the buildup of dysfunctional organelles since impaired mitochondria not only lose their capacity to generate ATP but also emit increased levels of ROS [54]. It is regulated by key proteins such as PINK1 and Parkin, which identify and tag damaged mitochondria for autophagic degradation [55, 56]. However, studies show that with aging, the efficiency of mitophagy declines significantly in muscle, heart, and brain tissues from aged mice and humans, in which PINK1 and Parkin levels are markedly reduced, with the accumulation of dysfunctional mitochondria [46]. Aligned with these findings, enhancing mitophagy has been shown to extend lifespan in mice and improve OXPHOS function in aged worms and mice [57, 58].

SIRT3 functions in mitophagy by deacetylating Forkhead box O3 (FOXO3), which activates the PINK1–Parkin pathway. This activation leads to the expression of Bcl-2 interacting protein 3 (Bnip3), microtubule-associated protein light chain 3 (LC3), DRP1, mitochondrial fission protein fission 1 (FIS1), and mitofusin 2 (MFN2), proteins essential for mitophagy and the regulation of mitochondrial fission and fusion [59]. As organisms age, the dyregulation of SIRT3 may affect these pathways and thus disturb mitophagy regulation and mitochondrial protein homeostasis.

During aging, the level of SIRT4 changes in a tissue-dependent manner. For example, SIRT4 is reported to be up-regulated in photo-aged human skin, while down-regulated in the liver from old mice [22, 40]. Under normal conditions, SIRT4 interacts with GTPase optic atrophy 1 (OPA1) to maintain the mitochondrial quality [20]. However, under mitochondrial stress, overexpression of SIRT4 increases mitochondrial mass and fusion, while reducing Parkin-regulated mitophagy, partly through its interaction with OPA1, ultimately resulting in a reduced capacity to eliminate dysfunctional mitochondria in HEK293 cell lines during aging [20]. For SIRT5, its role in mitophagy during aging is rarely reported. However, during starvation, the deletion of Sirt5 impaired mitochondrial elongation, leading to an increase in mitophagy. These findings suggest that Sirt5 functions as an energy sensor necessary for starvation-induced mitochondrial elongation and the prevention of autophagic degradation [24].

#### Mitochondrial sirtuins in fission and fusion

Like mitophagy, when stress surpasses the mtUPR’s capacity, mitochondrial membrane dynamics, including fission and fusion, are also engaged to manage the overload. By diluting and segregating damaged mitochondria, cells maintain homeostasis and enhance survival under stress via membrane dynamics [60]. Fission is primarily mediated by DRP1, while fusion is regulated by proteins, such as mitofusin 1 (MFN1), MFN2, and OPA1 [61]. Research indicates that the expression of these key proteins declines with age, leading to an accumulation of fragmented and dysfunctional mitochondria. In aged skeletal muscle, DRP1 levels are reduced, impairing the cell’s capacity to fragment and isolate damaged mitochondrial components, thereby hampering mitophagy [62]. Similarly, reduced MFN2 expression in aged cardiac tissue has been linked to mitochondrial fragmentation, compromised metabolic efficiency, and elevated ROS production, which collectively exacerbate age-related cardiac dysfunction [63].

SIRT3 directly deacetylates OPA1, a key regulator of mitochondrial fusion, thereby ensuring the proper balance between mitochondrial fusion and fission processes [17]. Nevertheless, SIRT3 attenuates doxorubicin-induced cardiotoxicity by inhibiting the NOD-like receptor protein 3 (NLRP3) inflammasome via mitophagy [64]. It has been proposed that SIRT3 plays a key role in maintaining the balance between mitochondrial division and fusion in neurons, helping to prevent or slow neuronal axon damage and degeneration caused by mitochondrial fragmentation and the resulting insufficient ATP supply [38].

By contrast, activation of SIRT4 has been found to regulate mitochondrial dynamics by inhibiting ERK-mediated phosphorylation of the pro-fission factor DRP1, thereby suppressing its activity and subsequently reducing mitochondrial fission to inhibit malignancy progression of non-small-cell lung cancer [21]. Furthermore, SIRT5 overexpression enhances the levels of mitochondrial fusion markers such as MFN2 and OPA1, indicating SIRT5’s involvement in regulating autophagy through mitochondrial quality control [23]. Although current research on the role of SIRT4 and SIRT5 in mitochondrial quality control during aging or age-related diseases is limited, the findings above suggest that SIRT4 and SIRT5 likely play an important role in regulating mitochondrial quality in the context of aging.

### Mitochondrial sirtuins in aging-related compromised membrane integrity

Under normal conditions, the inner mitochondrial membrane maintains selective permeability to preserve the mitochondrial membrane potential (Δψ_m_), which is essential for ATP production and energy homeostasis. This stability is partly controlled by the mitochondrial permeability transition pore (mPTP), which typically opens to regulate the ion and ROS levels within mitochondria [65]. However, aging disrupts mPTP regulation, leading to prolonged pore openings that destabilize mitochondrial homeostasis and promote cellular aging [66]. Increased mPTP opening results in a loss of Δψ_m_, allowing uncontrolled calcium influx and mtROS, which are particularly damaging to high-energy-demand tissues such as the brain and liver [67].

Oxidative stress plays a major role in this aging-related dysregulation, as mitochondria accumulate mtROS over time, which promotes mPTP opening via oxidizing SH residues on core components of mPTP, such as ATP synthase (F1F0), adenine nucleotide translocase (ANT), mPTP-associated protein cyclophilin D (CypD), and the outer membrane protein voltage-dependent anion channels (VDAC) [67]. Additionally, the accumulation of oxidized lipids, particularly peroxidized cardiolipin and phosphatidylethanolamine, destabilizes the mitochondrial membrane by altering fluidity and compromising electron transport, which further amplifies ROS production in a cycle of oxidative damage [68]. Thus, age-related increases in mitochondrial membrane permeability from persistent mPTP opening, oxidative stress, and lipid peroxidation perpetuate a cycle of mitochondrial dysfunction, impairing tissue health. Targeting mPTP regulation and mitigating oxidative stress are promising therapeutic strategies for delaying these permeability-related mitochondrial changes and preserving cellular function with age [69].

SIRT3 was reported to deacetylate CypD, a key regulator of the mPTP, to maintain the mitochondrial membrane permeability. SIRT3 knockout mice exhibit age-dependent mitochondrial dysfunction due to prolonged pore opening, displaying cardiac hypertrophy, fibrosis, and increased sensitivity to cardiac stress [70]. Additionally, SIRT3 has been proposed to restore mitochondrial membrane potential via interacting with ATP synthase in response to stress, suggesting that the decline in SIRT3 levels with aging may impair the ability of mitochondria to recover membrane potential, leading to diseases linked to mitochondrial homeostasis, such as Parkinson’s disease [71]. Furthermore, inhibition of SIRT3 using siRNA led to the acetylation of cytochrome c oxidase-1 (COX-1), a subunit of mitochondrial complex IV, disrupting of mitochondrial membrane potential [72].

Overexpression of SIRT4 enhanced mitochondrial membrane potential and suppressed the expression of apoptosis-related proteins, including NADPH oxidase 1 (NOX1), BCL2-associated X (BAX), and phosphorylated p38. Furthermore, SIRT4 overexpression markedly reduced the inflammatory response, suggesting that SIRT4 plays a protective role in preventing early pathological mechanisms underlying diabetic nephropathy [73]. Consistently, SIRT5 depletion in human proximal tubular epithelial cells led to reduced ATP production, decreased mitochondrial membrane potential, and induced mitochondrial fragmentation, indicating its protective function in maintaining membrane permeability [74]. The specific mechanisms by which SIRT4 and SIRT5 contribute to mitochondrial membrane integrity remain to be explored, but it is hypothesized that they protect the mitochondrial membrane by counteracting ROS. The role of mitochondrial sirtuins in ROS regulation will be discussed in detail in the next section. Overall, they play a critical role in maintaining mitochondrial membrane integrity during aging.

### Mitochondrial sirtuins in aging-associated elevated ROS production

Aging is closely associated with increased production of ROS within mitochondria, which leads to cumulative oxidative damage and contributes to the decline in cellular function. Mitochondria are a primary source of ROS, which are generated as byproducts of the ETC during oxidative phosphorylation [75]. With age, inefficiencies in ETC function led to electron leakage, particularly at Complexes I and III, resulting in higher ROS levels [76]. These excess ROS species, such as superoxide and hydrogen peroxide, can damage mtDNA, proteins, and lipids, further impairing ETC function and creating a feedback loop of escalating oxidative stress and mitochondrial dysfunction [77]. Studies indicate that this process is exacerbated by a decline in antioxidant defenses with age, including key enzymes like SOD and glutathione peroxidase, leading to reduced capacity to neutralize ROS [77]. For example, in multiple age-related diseases, such as cardiac diseases, diabetes, and neurodegenerative diseases, elevated ROS levels have been linked to increased mPTP opening, reducing ATP production, and accelerating degeneration [66]. Moreover, ROS-driven lipid peroxidation disrupts the mitochondrial membrane’s fluidity and structure, worsening electron leakage and amplifying oxidative damage [66]. Collectively, this self-perpetuating cycle of ROS production and mitochondrial damage underlies much of the cellular decline associated with aging and has been implicated in age-related diseases, such as neurodegeneration, sarcopenia, and cardiovascular dysfunction [66]. By contrast, the rate of mitochondrial ROS production is reduced in long-lived animal species [76].

SIRT3 plays a key role in mitigating ROS production and oxidative stress through two major mechanisms. First, it regulates ROS levels in the mitochondria by influencing the TCA cycle, particularly by modulating PDH activity. Second, SIRT3 deacetylates and activates key antioxidant enzymes, such as MnSOD and isocitrate dehydrogenase 2 (IDH2), which neutralize free radicals and reduce ROS generation [78, 79]. By inhibiting the activity of mitochondrial respiratory complexes I through V, SIRT3 further limits ROS production [80]. Additionally, SIRT3 targets the transcription factor FOXO3, which governs various stress response pathways, further enhancing cellular resilience to oxidative damage [81]. SIRT3 also forms a regulatory loop with PGC-1α, promoting MnSOD expression and contributing to the control of ROS levels in cells [79].

Unlike SIRT3, SIRT4 has been reported to increase ROS levels. Upregulation of endogenous SIRT4 expression reduces mitochondrial membrane potential, elevates mtROS levels, and induces changes in mitochondrial morphology, leading to increased aggregation [22]. Furthermore, SIRT4 levels are elevated in oocytes from aged mice [82]. By contrast, knocking down SIRT4, which decreases cellular ROS levels, partially rescues the impaired oocyte phenotypes in aged mice [83]. Similarly, in mouse hearts, SIRT4 inhibits SIRT3-mediated SOD2 activation potentially by competitively interacting with Mn-SOD for binding to SIRT3, leading to higher ROS levels and heart failure [84].

Similar to SIRT3, SIRT5 plays an important role in reducing ROS levels. SIRT5 activates IDH2 by desuccinylation, while SIRT3 achieves the same effect through deacetylation [85]. Furthermore, in the pentose phosphate pathway, SIRT5 removes glutaryl groups from glucose-6-phosphate dehydrogenase (G6PD) via deglutarylation, enhancing NADPH production and promoting the reduction of oxidized glutathione in mice [85]. Additionally, in lung tumor cells, cytosolic SIRT5 desuccinylates and activates copper-zinc superoxide dismutase (Cu/ZnSOD), leading to decreased ROS levels [86]. In a Parkinson’s disease mouse model induced by oxidative stress, SIRT5 deficiency resulted in severe neuronal degeneration and motor deficits, whereas SIRT5 knockout mice showed no significant phenotype under normal physiological conditions [87]. Overall, both SIRT5 and SIRT3 protect cells from oxidative stress, while SIRT4 exacerbates it.

### Mitochondrial sirtuins in aging-related damage of mtDNA

mtDNA is the small, circular DNA found in mitochondria, inherited maternally and distinct from nuclear DNA. It contains 37 genes essential for mitochondrial function, particularly in energy production through oxidative phosphorylation [88]. Unlike nuclear DNA, mtDNA replication occurs continuously and independently of the cell cycle. Due to its limited repair mechanisms, mtDNA is prone to accumulating base errors over time. As a result, mutations in mtDNA progressively accumulate in somatic cells throughout an organism’s life, leading to increased heteroplasmy—the coexistence of normal and mutated mtDNA within the same cell [89]. When heteroplasmy exceeds a critical threshold, it can have harmful physiological effects, driving aging and diseases such as impaired glucose metabolism, cognitive decline, and reduced lifespan, as demonstrated in mouse studies [90]. In addition, mtDNA is located in close proximity to the ETC, where ROS are generated as byproducts of oxidative phosphorylation. The exposure of mtDNA to elevated ROS levels over time results in oxidative damage to nucleotide bases, which can lead to severe point mutations and deletions, compromising the integrity of the ETC in numerous types of cancer cells [91]. During aging, mtDNA leaks out of the organelle, causing activation of inflammasomes or cytosolic DNA sensors, respectively [92]. The accumulation of mtDNA mutations leads to the destabilization of respiratory chain complexes, reduced turnover of the organelle, and changes in mitochondrial dynamics.

Mitochondrial sirtuins play crucial roles in maintaining the integrity and function of mtDNA mainly through regulating mtDNA repairment and ROS production [92]. In detail, in *Sirt3* knockout mice, there is increased mtDNA damage and cardiac hypertrophy, whereas SIRT3 overexpression protects mtDNA through maintaining levels of oxoguanine-DNA glycosylase-1 (OGG1), a key enzyme that repairs mtDNA damage, thus highlighting SIRT3’s crucial role in safeguarding mitochondrial integrity under Doxo treatment [93]. Additionally, SIRT3 has been shown to protect mitochondria from oxidative stress by deacetylating SOD2, enhancing its antioxidant activity, and reducing mtROS in mouse and human articular chondrocytes [94]. SIRT4 and SIRT5 are less studied in regulating mtDNA repair or protection. SIRT5 may contribute to mtDNA maintenance through preventing excessive oxidative stress that can damage mtDNA, while SIRT4 and SIRT5 may also interact with or complement the functions of SIRT3, which directly deacetylates and activates enzymes involved in protecting mtDNA.

Given SIRT3’s crucial role in maintaining mtDNA, pharmacologic SIRT3 inducers like resveratrol, viniferin, and honokiol have been shown to reduce oxidant-induced mtDNA damage in alveolar epithelial cells *in vitro* [95]. Notably, honokiol’s protective effects are specifically SIRT3-dependent. Therefore, targeting SIRT3 to preserve mtDNA presents a promising therapeutic approach for patients with idiopathic pulmonary fibrosis and other forms of pulmonary fibrosis.

### Mitochondrial sirtuins in aging-related metabolic imbalance

Aging and age-related diseases are closely associated with disruptions in the balance between energy supply and demand [96]. This imbalance can potentially be mitigated through various strategies, including regular physical activity, calorie restriction (CR), and the use of therapeutic agents such as metformin, resveratrol, and rapamycin [97]. Excessive calorie intake downregulates OXPHOS and antioxidant defenses, leaving the ETC in a chronically reduced state that promotes ROS production. By contrast, CR alleviates this by reducing ROS production, enhancing OXPHOS and antioxidant defenses, preventing age-related diseases, and extending lifespan [98]. CR significantly alters key metabolic regulatory pathways, including PI3K/AKT, mTOR, AMPK, and sirtuins. These pathways form the core metabolic signaling axis, highlighting the central role of metabolism in lifespan extension [99, 100].

SIRT3 has numerous deacetylase targets, including acetyl-CoA synthetase 2 (AceCS2), LKB1, ornithine transcarbamoylase (OTC), and FOXO3A, among others [15]. Interestingly, many of these targets are also activated during CR, pointing to a potential nutrient-sensing function for SIRT3 [101]. First, SIRT3 plays a crucial role in nutrient sensing by maintaining ATP levels through regulation of the ETC. In SIRT3 knockout mice, ATP levels in several organs are reduced by over 50%, as SIRT3 deacetylates and activates key ETC components like NADH:ubiquinone oxidoreductase subunit A9 (NDUFA9), succinate dehydrogenase complex flavoprotein subunit A (SDHA), and oligomycin sensitivity conferral protein (OSCP) [102]. SIRT3 also promotes the generation of acetyl-CoA by deacetylating the PDC under normal conditions, linking glycolysis to the Krebs cycle. During fasting, SIRT3 activates AceCS2, supporting acetyl-CoA production under low-carbohydrate, high-fat conditions, highlighting its regulatory role in metabolism under varying nutritional states [103]. Furthermore, SIRT3 is integral to metabolic control by promoting fatty acid oxidation and amino acid catabolism. It removes acetyl groups from key fatty acid oxidation enzymes, including long-chain acyl-CoA dehydrogenase (LCAD), medium-chain acyl-CoA dehydrogenase (MCAD), and very long-chain acyl-CoA dehydrogenase (VLCAD), while also controlling the conversion of acetyl-CoA into ketone bodies under fasting conditions [104]. Additionally, SIRT3 enhances the urea cycle by deacetylating OTC, thereby supporting amino acid metabolism [105].

SIRT4 primarily functions as an NAD^+^-dependent ADP-ribosyltransferase, contrasting with the deacetylase roles of SIRT3 and SIRT5 [106]. It negatively regulates metabolic pathways, such as glutamine catabolism, fatty acid oxidation, and amino acid catabolism. SIRT4 inhibits GDH activity via ADP-ribosylation, thereby reducing glutamine metabolism, while SIRT3 enhances GDH activity through deacetylation [107]. Additionally, SIRT4 suppresses fatty acid oxidation by inhibiting malonyl-CoA decarboxylase (MCD) and downregulating PPARα, a key regulator of fatty acid catabolism [108].

SIRT5, unlike SIRT3 and SIRT4, exhibits weak deacetylase activity but is known for catalyzing demalonylation, desuccinylation, and deglutarylation of mitochondrial enzymes involved in metabolic pathways, such as glycolysis, fatty acid oxidation, and the urea cycle [25]. While SIRT5 knockout mice show no significant metabolic abnormalities under basal conditions, under CR, SIRT5 deficiency reduces the activity of carbamoyl phosphate synthetase (CPS1), affecting amino acid catabolism [109]. Additionally, SIRT5 loss under starvation induces mitochondrial fission and mitophagy. SIRT5 regulates glycolysis by demalonylating enzymes like GAPDH and desuccinylating pyruvate kinase M2 (PKM2) [110]. It also modulates fatty acid oxidation through the desuccinylation of enzymes, such as VLCAD, working with SIRT3 to promote this process, particularly during fasting, when SIRT5 enhances ketone body formation [111].

CR is one of the most robust interventions for lifespan extension in mammals [112]. Of note, mitochondrial sirtuins play important roles in regulating metabolic pathways during CR, including enhancing fatty acid oxidation, promoting ketone body production, and reducing oxidative stress, all of which contribute to mitigating aging-associated phenotypes [112]. It has been displayed that CR upregulates SIRT3 and SIRT5, while downregulating SIRT4, highlighting their opposing roles in metabolism during CR. SIRT3 and SIRT5 promote fatty acid oxidation and ketone body production, whereas SIRT4 inhibits GDH activity through ADP-ribosylation [107]. In contrast, a high-fat diet (HFD) has the opposite effect, reducing SIRT3 expression and leading to metabolic disorders like obesity and insulin resistance. Although SIRT3 generally protects against HFD-induced conditions, recent studies suggest it may also promote tumor formation under HFD, indicating its role is context-dependent [113].

### Mitochondrial sirtuins in aging-related disrupts of mitochondrial–nuclear crosstalk

More than 95% of mitochondrial proteins are encoded by nuclear DNA. As a result, mitochondrial function is highly reliant on the nucleus responding to signals from the mitochondria, a process known as “retrograde signaling” [114]. Triggers for the retrograde response include changes in the ATP/ADP ratio, mitochondrial membrane potential disruption, ROS, and overall cellular stress [115]. Disruption of the mitochondrial–nuclear communication with age contributes to cellular dysfunction and the decline of tissue health. For example, telomere dysfunction results in cellular growth arrest and senescence through p53-mediated pathway and impaired mitochondrial biogenesis via affecting PGC-1α [116]. In addition, mtUPR triggers nuclear transcriptional changes in response to mitochondrial stress, while aging attenuates mtUPR signaling, contributing to protein aggregation and impaired mitochondrial function [46]. Moreover, humanin, a 24-amino acid peptide encoded within the mitochondrial 16S ribosomal RNA, interacts with cell surface receptors to play a protective role by inhibiting apoptosis, reducing inflammation, and mitigating oxidative stress across various aging models [117].

As described above, mitochondrial sirtuins play a critical role in metabolic regulation, while metabolic byproducts, in turn, influence gene expression in the cell nucleus. Studies suggest that ATP concentration serves as a key trigger for the mitochondria-to-nucleus retrograde response in yeast [118]. Moreover, the interplay between metabolism and epigenetics is increasingly recognized as a critical factor in aging and cellular homeostasis. Key metabolites such as acetyl-CoA, S-adenosylmethionine (SAM), and α-ketoglutarate (αKG) serve as essential cofactors and substrates for epigenetic modifications, linking mitochondrial function to nuclear gene regulation.

Acetyl-CoA powers the TCA cycle to generate ATP aerobically while also serving as a fundamental precursor for cholesterol, lipids, amino acids, and other essential biomolecules needed for cellular growth. Additionally, it acts as a substrate for histone acetyltransferases (HATs), which acetylate histone tails to modulate chromatin structure and regulate gene expression in eukaryotes [12, 119]. SIRT3 promotes acetyl-CoA production by modulating AceCS2, which converts acetate into acetyl-CoA, thereby modulating histone acetylation and gene expression [120]. SAM, the primary methyl donor for histone and DNA methylation, plays a crucial role in epigenetic regulation. SIRT4 indirectly influences gene expression by regulating SAM levels through its interaction with MAT2A, a key enzyme responsible for SAM generation. Loss of SIRT4 activates MAT2A via ADP-ribosylation, leading to increased SAM levels and dynamic regulation of gene expression, which promotes the high proliferation rate of tumor cells [121]. αKG, a cofactor for DNA and histone-demethylating enzymes, links metabolic processes to epigenetic regulation. SIRT5 indirectly affects gene expression by regulating αKG production through its desuccinylase and demalonylase activities, which influence TCA cycle enzymes. For example, changes in αKG levels, regulated by SIRT5, can alter histone demethylation and gene expression in brown adipocytes [122].

**Figure 4.**
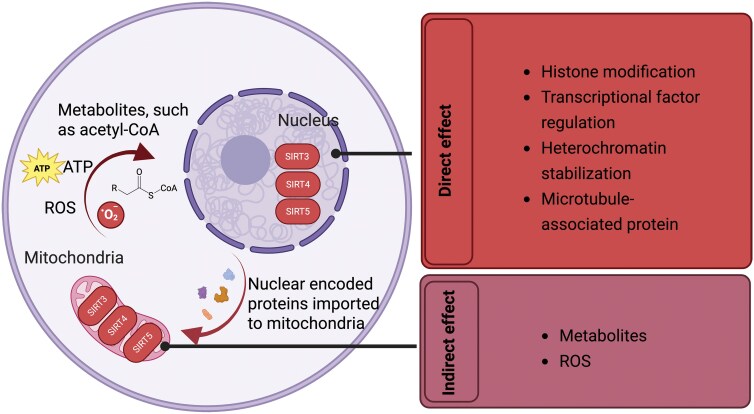
**Mitochondrial sirtuins in mitochondrial–nuclear crosstalk.**Mitochondrial sirtuins display pivotal roles in mitochondrial–nuclear crosstalk through both direct and indirect mechanisms. Directly, these sirtuins can translocate to the nucleus under certain conditions to regulate transcription and maintain epigenomic stability. Indirectly, they influence nuclear gene expression by modulating metabolic intermediates, such as NAD^+^ and acetyl-CoA, or by regulating ROS levels, thereby affecting redox-sensitive transcription factors.

In addition to regulating metabolism to indirectly influence gene expression, mitochondrial sirtuins have also been found in the cytoplasm and nucleus, where they directly participate in genomic and epigenomic regulation. While mitochondrial sirtuins can influence genomic and epigenomic stability through the regulation of metabolism and ROS [123, 124], their roles in metabolic and ROS control have already been discussed in the previous sections. Here, we will focus on the research concerning the direct role of mitochondrial sirtuins in regulating the genome and epigenome within the nucleus (Fig. 4).

SIRT3 has been demonstrated to possess histone deacetylase activity in both *in vitro* and *in vivo* models to maintain genomic integrity [125–128]. In addition, SIRT3 has also been shown to deacetylate non-histone proteins, such as Ku70, leading to the sequestration of the proapoptotic protein BAX in the nucleus [129]. In cardiac hypertrophy models, SIRT3 has been shown to regulate the transcription factor FOXO3A through deacetylation, promoting its translocation from the mitochondria to the nucleus. This process enhances the expression of antioxidant enzymes, illustrating the intracellular communication between the nucleus and mitochondria [130]. Moreover, studies have shown that SIRT3 is present in the nucleus, functioning to reinforce heterochromatin structure and delay senescence in human mesenchymal stem cells [131]. These findings highlight the importance of nuclear sirtuins in maintaining genomic stability during cellular stress, which is crucial for preventing aging.

Most SIRT4 and SIRT5 are localized in the mitochondrial matrix, with a fraction also found in the nucleus [132, 133]. A recent study found that SIRT4 functions as a microtubule-associated protein, regulating microtubule dynamics and cell cycle progression. SIRT4 overexpression reduces acetylated α-tubulin levels and delays mitosis under cellular stress [134]. Another study showed that SIRT5 plays a critical role in maintaining histone acetylation and methylation patterns in melanoma cells, thereby ensuring proper gene expression. Its deficiency results in impaired histone modifications and decreased expression of oncogenes such as MITF and c-MYC [135]. The functions of SIRT4 and SIRT5 within the nucleus remain relatively unexplored and warrant further investigation.

## Mitochondrial sirtuins in tissue aging and aging-related disease

### Mitochondrial sirtuins’ tissue-specific effects in aging

The tissue-specific effects of mitochondrial sirtuins are increasingly recognized as key factors in determining organ health and aging trajectories. SIRT3 is preferentially expressed in organs with high oxidative activity, including the brain, heart, liver, brown adipose tissue, and muscle. Conversely, its presence is minimal in less metabolically active areas, such as white adipose tissue, lungs, spleen, thymus, pancreas, and the small intestine [136]. By contrast, the levels of SIRT4 were highest in the liver and islets, but were found to be low in the heart, skeletal muscle, and pancreas in mice [137]. SIRT5 is mainly enriched in lung, stomach, liver, and heart [138]. In the brain, SIRT3 is essential for maintaining neuronal energy homeostasis and protecting against oxidative stress, which is particularly important for post-mitotic cells that rely heavily on mitochondrial function. Age-related declines in SIRT3 activity have been linked to neurodegenerative diseases, such as Alzheimer’s and Parkinson’s, where mitochondrial dysfunction and oxidative damage are hallmarks [136]. In skeletal muscle, SIRT3 and SIRT4 regulate mitochondrial biogenesis, fatty acid oxidation, and ATP production, which are critical for maintaining muscle strength and endurance [139, 140]. Dysregulation of these sirtuins contributes to age-related sarcopenia, characterized by muscle atrophy, reduced regenerative capacity, and increased frailty.

In the liver, mitochondrial sirtuins play a central role in metabolic regulation, with SIRT3 and SIRT5 being particularly important. SIRT3 regulates the TCA cycle and fatty acid oxidation, while SIRT5 modulates ammonia detoxification and ketogenesis [19, 23]. Dysregulation of these sirtuins is associated with metabolic disorders such as non-alcoholic fatty liver disease and insulin resistance, which are more prevalent with aging. Similarly, in adipose tissue, SIRT4 influences lipid metabolism and insulin sensitivity, and its downregulation is linked to age-related metabolic syndrome and type 2 diabetes (T2D) [141]. The heart is another organ where mitochondrial sirtuins, particularly SIRT3, play a critical role in maintaining function and resilience. SIRT3 regulates mitochondrial dynamics, oxidative stress responses, and energy production in cardiomyocytes, which are essential for sustaining cardiac output and preventing hypertrophy and fibrosis. Age-related declines in SIRT3 activity are associated with increased susceptibility to cardiovascular diseases, such as heart failure and arrhythmias [142]. In contrast, in the kidneys, SIRT3 protects against age-related inflammation, fibrosis, and oxidative damage, which are key contributors to renal dysfunction. The loss of SIRT3 in the kidneys exacerbates age-related decline in filtration capacity and increases the risk of chronic kidney disease [143].

The heterogeneity of aging phenotypes across different organs can be attributed to the differential expression, regulation, and functional roles of mitochondrial sirtuins. For instance, the brain and heart, which are highly dependent on mitochondrial function, exhibit pronounced age-related declines when sirtuin activity is compromised. In contrast, tissues like the liver and adipose tissue, which are more metabolically active, experience age-related dysregulation of sirtuins that leads to metabolic imbalances and systemic effects [144]. This variability in sirtuin function across tissues explains why aging manifests differently in each organ, with some tissues showing more severe functional decline than others. Understanding these tissue-specific mechanisms is crucial for developing targeted interventions to mitigate the effects of aging. By restoring mitochondrial sirtuin activity in a tissue-specific manner, it may be possible to address the unique aging challenges faced by each organ, thereby improving overall healthspan and reducing the burden of age-related diseases.

### Mitochondrial sirtuins in cardiovascular diseases

**Figure 5.**
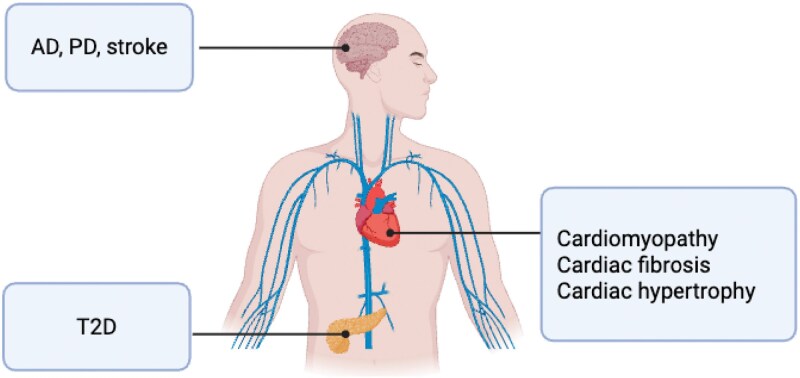
**Mitochondrial sirtuins dysfunction in age-related diseases.**SIRT3, SIRT4, and SIRT5 are implicated in various age-related diseases due to their critical roles in maintaining mitochondrial function and metabolic homeostasis. Dysregulation of these sirtuins has been linked to metabolic disorders such as T2D, cardiovascular diseases, and neurodegenerative conditions. The dysfunction of these sirtuins exacerbates mitochondrial decline, contributing to the progression of aging-related diseases.

SIRT3, SIRT4, and SIRT5 are implicated in various age-related diseases due to their critical roles in maintaining mitochondrial function and metabolic homeostasis (Fig. 5). Among these age-related diseases, cardiovascular diseases refer to a group of disorders affecting the heart and blood vessels, including conditions such as coronary artery disease, heart failure, arrhythmias, and stroke, which are a major cause of morbidity and mortality, particularly among the elderly [67, 145, 146]. SIRT3 knockout mice exhibit reduced cardiac mitochondrial mass, dysfunctional mitochondrial networks, and impaired calcium homeostasis, leading to compromised cardiac contraction, reduced ATP synthesis, and mitochondrial respiration dysfunction. These conditions contribute to a shorter lifespan and age-related cardiac pathology in mice [147]. In contrast, overexpressing SIRT3 protects against doxorubicin-induced cardiomyopathy and mitochondrial-induced apoptosis by deacetylating and activating OPA1, preserving mitochondrial cristae structure and preventing cytochrome C release into the cytosol [93, 129]. In SIRT3 knockout human cardiomyocytes, altered mitochondrial clustering is observed, with fewer and morphologically abnormal perinuclear mitochondria [148]. This clustering is crucial for ATP production and mitochondrial–nuclear energy communication. Mitochondrial dysfunction in cardiomyocytes impairs bioenergetics and reduced cell survival, contributing to age-related cardiac fibrosis [149, 150].

By contrast, SIRT4 plays a dual role in cardiac health. In cardiac hypertrophy, SIRT4 can be detrimental by increasing ROS levels through its interaction with SIRT3 and MnSOD [84]. However, SIRT4 also has protective roles in ischemic cardiomyopathy and antineoplastic therapy-induced cardiac injury [124]. It has also been shown that SIRT4 or SIRT5 can ameliorate IR-induced cardiomyocyte senescence via deacetylation [151]. In addition, SIRT5 plays a crucial role in activating ECHA, a key enzyme involved in fatty acid catabolism in the myocardium, through desuccinylation. In line with this, SIRT5 knockout mice exhibit reduced fatty acid oxidation and impaired energy production in the heart during exercise or fasting, ultimately leading to the development of hypertrophic cardiomyopathy [111]. In comparison, overexpression of SIRT5 improved cardiac function and decreased fibrosis in mice [111]. Together, SIRT3, SIRT4, and SIRT5 play complementary roles in maintaining cardiac health by preserving mitochondrial function and energy metabolism. SIRT3 and SIRT5 primarily protect against mitochondrial dysfunction and energy deficits, while SIRT4 exhibits context-dependent effects, offering both protective and detrimental roles depending on the condition. Their combined actions highlight the intricate coordination required to mitigate cardiovascular diseases.

### Mitochondrial sirtuins in T2D

T2D is a chronic metabolic disease often associated with aging and characterized by high blood sugar levels. Researchers have found that SIRT3 activation enhances insulin sensitivity through the regulation of insulin signaling, whereas SIRT3 inhibition exacerbates insulin resistance [152]. SIRT3 was also found to reduce cognitive defects in diabetic mice via preventing abnormal mitochondria-associated membranes (MAMs) formation [153]. By contrast, SIRT4 again plays a dual role in T2D. Prior studies revealed that SIRT4 directly binds to glutamate GDH and inhibits its activity, partially through ADP-ribosylation. This inhibition suppresses glutamine metabolism, reduces ATP production, and decreases insulin secretion in pancreatic beta cells. Reducing SIRT4 expression stimulates GDH activity, thereby improving the ability of β-cells to secrete insulin in response to changes in blood sugar levels in mice [141, 154]. However, in contrast to previous findings, SIRT4 knockout mice initially exhibited enhanced insulin secretion, but this eventually led to glucose intolerance and insulin resistance. SIRT4 modulates insulin sensitivity by activating methylcrotonyl-CoA carboxylase 1 (MCCC1) through the removal of dicarboxyacyl-lysine modifications. This activation promotes leucine catabolism, ultimately leading to reduced insulin secretion from the pancreas [137]. These findings suggest that the function of SIRT4 in T2D is context-dependent and tissue-specific, requiring more studies to reveal the detailed mechanism [155]. Of note, SIRT5 expression is elevated in T2D patients and pancreatic β-cells, which is positively associated with age and blood glucose levels. Furthermore, the downregulation of SIRT5 promoted the secretion of insulin, suggesting that targeting SIRT5 may offer a novel therapeutic approach for T2D [156]. In summary, SIRT3 primarily enhances insulin sensitivity and protects against mitochondrial dysfunction, while SIRT4 exhibits dual, context-dependent roles, influencing both insulin secretion and glucose tolerance. SIRT5, on the other hand, is positively associated with T2D progression and may serve as a potential therapeutic target.

### Mitochondrial sirtuins in neurodegenerative diseases

Metabolic homeostasis and ROS level regulation are crucial for maintaining the health of neurons [157–159]. As a result, the malfunction of mitochondrial sirtuins, which disrupts these processes, is implicated in multiple neurodegenerative diseases, including Parkinson’s, Alzheimer’s, dementia, and epilepsy. Cortical samples from AD patients exhibit lower levels of Sirt3 mRNA expression compared to those from healthy individuals [160]. In addition, curcumin, known for its neuroprotective properties, has been shown to attenuate Aβ-induced neuronal metabolic dysfunction and improve cognitive function in an AD mouse model by upregulating SIRT3, indicating that SIRT3 plays a neuroprotective role in AD, potentially by modulating Aβ-related pathways [161]. Furthermore, previous studies have shown that SIRT3 protects neurons by maintaining mitochondrial energy metabolism in Parkinson’s disease (PD). Moreover, SIRT3 protects dopaminergic neurons by deacetylating SOD2 and ATP synthase β-subunit, thereby reducing oxidative stress and preventing cell death. Theacrine, a purine alkaloid, has been shown to activate SIRT3 and inhibit ROS production, ultimately protecting dopaminergic neurons from apoptosis, suggesting SIRT3’s important roles in PD [162–164].

Like SIRT3, SIRT4 also has a neuroprotective role. For example, SIRT4 deficiency results in lower levels of glutamate transporter proteins on the neuronal membrane, impairing glutamate uptake and rendering neurons more vulnerable to excitotoxic damage [165]. Consistently, SIRT5 plays a neuroprotective role in AD by mitigating neuronal injury. Studies have shown that SIRT5 downregulation and decreased autophagy contribute to AD progression in mouse models [166]. SIRT5 overexpression can counteract these effects, likely by increasing SOD activity, reducing ROS levels, and inhibiting microglia and astrocyte activation. These findings suggest that targeting SIRT5 may be a potential therapeutic strategy for AD [166]. Together, these mitochondrial sirtuins form an interconnected network crucial for combating neurodegenerative diseases. SIRT3 primarily safeguards neurons by maintaining mitochondrial energy metabolism and reducing oxidative stress, while SIRT4 regulates glutamate transport to prevent excitotoxic damage. SIRT5 mitigates neuronal injury in AD by enhancing antioxidant defenses and modulating neuroinflammation.

## Therapeutic interventions targeting mitochondrial sirtuins

**Figure 6.**
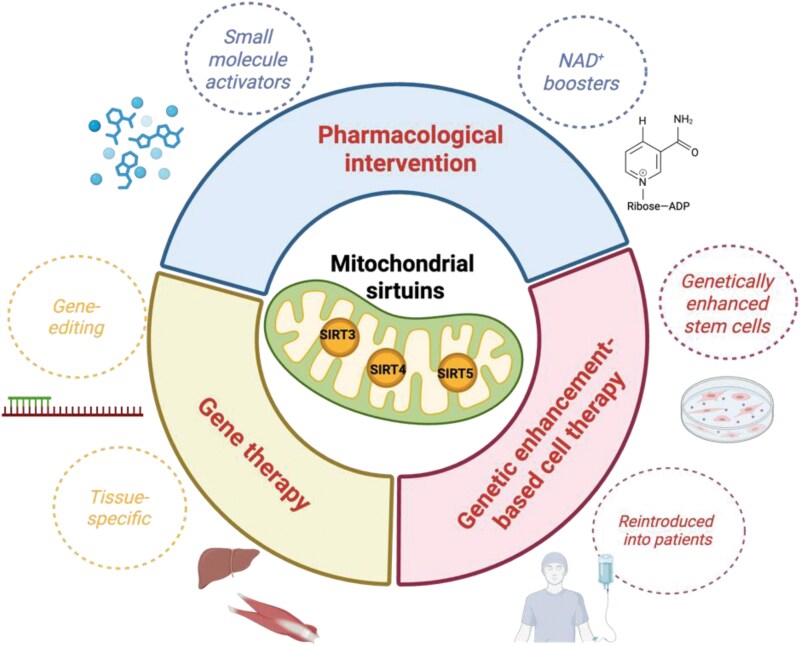
**Therapeutic strategies targeting mitochondrial sirtuins.**Effective interventions and treatment strategies targeting mitochondrial sirtuins through pharmacological interventions, gene therapy, and genetic enhancement-based cell therapy approaches. These interventions targeting mitochondrial sirtuins represent a promising frontier in aging research, with the potential to transform the treatment of age-related diseases and improve healthspan.

Therapeutic strategies targeting mitochondrial sirtuins fall broadly into three categories: pharmacological interventions, gene therapy, and genetic enhancement technologies (Fig. 6). As aging and age-related diseases are increasingly linked to mitochondrial dysfunction and dysregulated sirtuin signaling, the development of these intervention modalities represents a significant advance in translational geroscience [167].

### Pharmacological interventions targeting mitochondrial sirtuins

Current pharmacological strategies focus on small-molecule activators and NAD^+^ boosters, which aim to enhance sirtuin activity and restore mitochondrial homeostasis [168]. For example, compounds like resveratrol and synthetic sirtuin-activating compounds (STACs) have shown potential in preclinical studies by activating SIRT1, which indirectly influences mitochondrial sirtuins through downstream signaling pathways [169]. However, direct activators of mitochondrial sirtuins remain limited, highlighting the need for more targeted drug development to address tissue-specific mitochondrial dysfunction in aging and disease.

One of the most promising approaches to enhance mitochondrial sirtuin activity is through boosting cellular NAD^+^ levels, as NAD^+^ is an essential cofactor for sirtuin function [168, 170]. NAD^+^ precursors, such as nicotinamide riboside (NR) and nicotinamide mononucleotide (NMN), have demonstrated efficacy in preclinical models by restoring mitochondrial sirtuin activity and improving metabolic health. For instance, NMN supplementation has been shown to enhance SIRT3 activity, leading to improved mitochondrial function and reduced oxidative stress in aged tissues [171]. Clinical trials are currently evaluating the safety and efficacy of these precursors in humans, with early results indicating improvements in markers of metabolic health and cardiovascular function. These findings underscore the potential of NAD^+^ boosting as a strategy to counteract age-related declines in mitochondrial sirtuin activity and mitigate associated pathologies.

Emerging strategies are also exploring the development of direct activators and modulators of mitochondrial sirtuins. Advances in structural biology and drug design have enabled the identification of compounds that specifically target SIRT3, SIRT4, and SIRT5. For example, small molecules, such as 2-APQC and C12, that enhance SIRT3 activity, have shown promise in protecting against cardiac hypertrophy, neurodegeneration, and metabolic disorders in animal models [172, 173]. Additionally, SIRT5 modulators are being investigated for their potential for metabolic and age-related diseases, such as T2D, AD, and cancer [174].

### Gene therapy targeting mitochondrial sirtuins

Unlike pharmacological agents that rely on systemic bioavailability and may suffer from limited tissue specificity, gene therapy enables direct delivery of functional sirtuin genes to affected organs [175]. This is especially relevant in post-mitotic tissues, such as skeletal muscle, heart, and brain, where mitochondrial decline strongly correlates with age-related pathology [176]. By using tissue-tropic vectors and regulated expression systems, gene therapy can achieve long-lasting and localized upregulation of sirtuins, offering disease-modifying potential rather than symptomatic relief. For instance, researchers rejuvenated blood vessels and extended lifespan in a Hutchinson-Gilford progeria model using vascular endothelium-targeted *SIRT7* gene therapy [177]. Another example comes from a study on primate cardiac aging, where silencing of *SIRT2*, a member of the sirtuin family, was identified as both a hallmark and driver of cardiac aging [178]. Loss of *SIRT2* led to cardiomyocyte senescence, inflammation, and hypertrophy, whereas intra-myocardial delivery of a *SIRT2*-expressing lentivirus effectively alleviated age-associated cardiac degeneration in mice [179].

A compelling example targeting mitochondrial sirtuins of this approach is the recent study by Zhao et al., which demonstrated the efficacy of *SIRT5* gene therapy in aged mice [180]. Using lentiviral vectors to express wild-type *SIRT5* in skeletal muscle, the authors showed significant improvements in muscle mass, physical endurance, and mitochondrial function. Mechanistically, SIRT5 desuccinylated TBK1 at lysine 137, leading to decreased TBK1 phosphorylation and suppression of the NF-κB-mediated inflammatory response. This axis was identified as a key driver of age-related muscle degeneration. Importantly, SIRT5 deficiency in human myotubes induced cellular senescence, inflammation, and oxidative stress, all of which were reversed upon re-expression of *SIRT5*. These findings highlight not only the therapeutic relevance of the SIRT5–TBK1 pathway but also establish gene therapy as a viable modality for reversing molecular and physiological hallmarks of aging at the tissue level [180].

Future directions may include dual gene delivery strategies, such as co-expressing NAD⁺ biosynthesis enzymes with sirtuins, or integrating mitochondrial targeting sequences to enhance protein localization. As mitochondrial dysfunction is a central hallmark of aging and chronic disease, gene therapy targeting mitochondrial sirtuins represents a precise, durable, and increasingly practical strategy for extending healthspan and combating age-associated functional decline.

### Genetic enhancement-based cell therapy targeting mitochondrial sirtuins

Genetic enhancement refers to the *ex vivo* modification of therapeutic cells to improve their viability, functionality, and resistance to hostile microenvironments before transplantation into patients [181, 182]. By genetically modifying mesenchymal stem cells (MSCs), neural stem cells (NSCs), or hematopoietic stem cells (HSCs) to overexpress mitochondrial sirtuins, researchers aim to create a new class of “sirtuin-enhanced” therapeutic cells with superior survival and anti-aging capacity. These cells can then be reintroduced into patients to repair damaged tissues, restore metabolic balance, and modulate inflammation. For instance, studies have demonstrated that overexpression of *SIRT3* in HSCs restores their regenerative capacity and reduces oxidative damage in aged bone marrow environments [183]. Similarly, *SIRT5* overexpression has been shown to enhance mitochondrial respiration and reduce inflammatory signaling in myogenic stem cells [180].

Recent advances in base editing and CRISPR/Cas9 technologies have made it feasible to perform precise, single-nucleotide-level editing of sirtuin genes or their regulatory elements in human stem cells [184]. For example, introducing stabilizing mutations into the NRF2 pathway or FOXO3 has been used to delay senescence in MSCs, and similar strategies could be applied to sirtuin enhancers or promoters to achieve constitutive or inducible activation of SIRT3 or SIRT5 [185, 186]. In the future, the genetic enhancement of stem cells with mitochondrial sirtuins may not only improve their intrinsic metabolic fitness but also enhance their paracrine effects, contributing to systemic rejuvenation. This strategy aligns with the broader goal of building transplantable, rejuvenated cellular units that can resist age-related degeneration and restore function in affected tissues.

Despite the progress, challenges remain in translating these therapeutic strategies into clinical practice. Issues such as drug specificity, bioavailability, and potential off-target effects need to be addressed to ensure the safety and efficacy of mitochondrial sirtuin-targeted interventions. Furthermore, the heterogeneity of aging phenotypes across tissues necessitates a personalized approach to treatment, considering the differential expression and function of mitochondrial sirtuins in various organs. Advances in systems biology and multi-omics technologies are expected to provide deeper insights into the tissue-specific roles of mitochondrial sirtuins, enabling the development of tailored therapies [187]. Overall, interventions targeting mitochondrial sirtuins represent a promising frontier in aging research, with the potential to transform the treatment of age-related diseases and improve healthspan.

## Conclusion and future perspective

A recent study highlighted the dual role of mitochondria as both an energetic hub and a biosynthetic center, emphasizing its critical importance in cellular stress responses [188]. Innovative interventions demonstrated that delivering exogenous mitochondria to aged, non-activated CD4^+^ T cells induced significant changes in the mitochondrial proteome, characterized by enhanced aerobic metabolism and reduced mitochondrial ROS. This approach effectively reversed aging-associated mitochondrial dysfunction, improved the functionality of aged CD4^+^ T cells, and protected recipient mice from infections [189]. These findings collectively underscore mitochondria’s potential as a pivotal functional regulator during aging and a therapeutic target for aging-related disease treatment.

Mitochondrial sirtuins, as key enzymic regulators, modulate multiple mitochondrial functions, including metabolism, mtROS, proteome balance, and mitochondrial-nuclear communication. The known targets of SIRT3 include key enzymes involved in fatty acid oxidation (LCAD), the citric acid cycle (IDH2, GDH, SDHA), electron transport (NADH dehydrogenase), pyruvate metabolism (PDHA), ATP synthesis, amino acid metabolism (OTC), mitochondrial protein synthesis (MRPL10), mitochondrial folding (CypD and Hsp10), and mitochondrial protein degradation (LONP1) [38]. SIRT3 can also translocate to the nucleus and act as a histone deacetylase, regulating the epigenetic landscape of various genes [131]. SIRT4 can maintain genomic stability and mitochondrial function, regulate energy metabolism, and modulate stem cell depletion and inflammatory responses via multiple enzymic modifications [108]. SIRT5 promotes glycolytic metabolism while inhibiting oxidative phosphorylation. It also plays a crucial role in fatty acid oxidation and ammonia detoxification. SIRT5 enhances cellular survival and reduces oxidative stress by increasing NADPH production to support GSH regeneration and upregulating SOD1 activity [86].

The age-related dysregulation of mitochondrial sirtuins can severely impair mitochondrial function through malfunctional metabolic control, elevated ROS production, and disturbed transcriptional or epigenomic regulation, thereby accelerating cellular senescence, tissue degeneration, and aging-related diseases. Despite their recognized importance, the specific functions and mechanisms of mitochondrial sirtuins in aging remain incompletely understood, particularly regarding how they interact or counteract each other across different tissues and contexts. To unlock their full clinical potential, it is crucial to conduct comprehensive investigations into their molecular mechanisms and biological roles, utilizing advanced animal models. Such studies will not only identify promising therapeutic targets but also evaluate potential adverse effects, paving the way for the development of novel [190] interventions. Addressing these critical questions will deepen our understanding of mitochondrial sirtuins in aging and facilitate innovative strategies to combat age-related diseases.
